# Chronic Cough, Dyspnea, and a Novel CCDC39 Variant: A Case Report of Heterotaxy Syndrome Without Cardiac Anomalies and Associated Primary Ciliary Dyskinesia

**DOI:** 10.7759/cureus.76408

**Published:** 2024-12-26

**Authors:** Jochen Juré, Patrick Alexander

**Affiliations:** 1 Cardiology, Universitair Ziekenhuis Gent, Gent, BEL; 2 Pulmonology, Algemeen Ziekenhuis Glorieux, Ronse, BEL

**Keywords:** heterotaxy syndrome (hs), kartagener syndrome (ks), polysplenia syndrome, primary ciliary dyskinesia (pcd), situs ambiguous

## Abstract

Heterotaxy syndrome is characterized by abnormal left-right arrangement of thoracoabdominal organs and is frequently associated with complex cardiac anomalies. However, cases with predominant extracardiac manifestations are increasingly recognized. This report describes a 20-year-old female of North African descent with consanguineous parentage, who presented with chronic cough and exertional dyspnea persisting over several years. Clinical examination, biochemical analyses, and vital signs were unremarkable, and there was no reported history of environmental exposures or tuberculosis. Pulmonary function testing revealed severe airway obstruction, reversible with bronchodilators. Imaging studies demonstrated a diffuse bronchiolitis pattern, an enlarged azygos vein, and polysplenia. Abdominal CT (computed tomography) revealed an interrupted inferior vena cava with azygos continuation, an enlarged left liver, a multinodular spleen, and distal pancreatic atrophy. Methicillin-resistant *Staphylococcus aureus* was identified in bronchoalveolar lavage, and the patient was treated with intravenous vancomycin. Further evaluations, including sinus CT, revealed bilateral frontal sinus aplasia, hypoplasia of other sinuses, and structural abnormalities such as the absence of uncinate processes. Nasal biopsy showed absent ciliary motility, and transmission electron microscopy revealed inner dynein arm defects and central apparatus abnormalities without outer dynein arm involvement. Genetic testing identified a novel homozygous c.2347_2351del (p.Phe783ThrfsTer3) PVS1 null variant in exon 17 of the *CCDC39* gene, associated with autosomal recessive primary ciliary dyskinesia-14. This case highlights the overlap between heterotaxy syndrome and primary ciliary dyskinesia, suggesting that the ciliary defect contributed to both the patient’s organ laterality defects and chronic respiratory symptoms. The findings underscore the importance of a comprehensive evaluation of structural and functional abnormalities and the role of genetic testing in managing atypical presentations of heterotaxy syndrome.

## Introduction

Heterotaxy syndrome is a rare congenital disorder characterized by abnormal left-right asymmetry of the thoracoabdominal organs. This condition often presents with significant cardiac anomalies; however, it can also manifest with isolated extracardiac features, including respiratory and gastrointestinal abnormalities [1]. While heterotaxy is traditionally associated with structural defects, emerging evidence suggests that functional impairments, particularly those related to ciliary dysfunction, may also play a crucial role in the syndrome's clinical presentation. One such dysfunction is observed in primary ciliary dyskinesia (PCD), where defects in the motility of cilia lead to recurrent respiratory infections and other complications. This case report explores the connection between heterotaxy syndrome and a novel PVS1 null variant in exon 17 of the *CCDC39* gene, offering insights into the pathophysiology and management of patients with non-cardiac manifestations of the disorder.

## Case presentation

**Figure 1:**
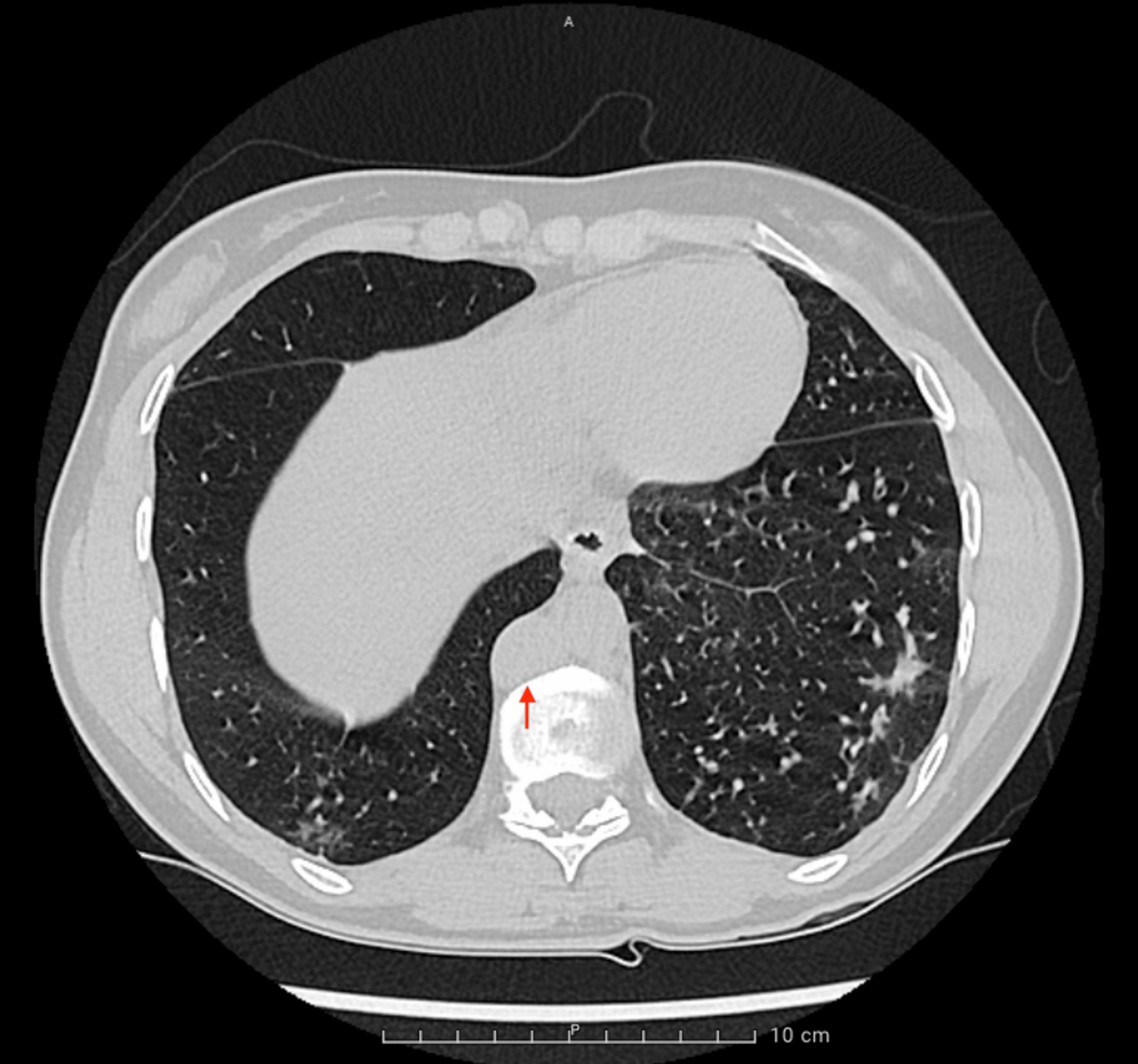
Chest CT demonstrating azygos continuation of the inferior vena cava (red arrow) and diffuse bronchiolitis pattern

A 20-year-old female patient of North African descent, born to first-cousin parents and no known siblings, was evaluated in the outpatient clinic due to persistent chronic cough and dyspnea on exertion that had been present for several years. Besides anosmia, the patient’s review of systems was otherwise unremarkable. Vital signs and clinical examination were within normal limits. The patient reported no history of allergies or potential exposure to environmental toxins or tuberculosis. Biochemical analysis results were within normal limits (Table 1). Pulmonary function testing revealed the presence of severe airway obstruction, which was reversible with the use of bronchodilators. Despite initiating therapy with saline nasal irrigation and intranasal corticosteroids, the patient’s symptoms persisted. Further evaluation with a chest CT (computed tomography) scan showed a diffuse bronchiolitis pattern and revealed a persistent caval vein and a plump azygos vein (Figure 1). A sputum culture and bronchoalveolar lavage showed the presence of methicillin-resistant *Staphylococcus aureus *(MRSA) (Table 2). The patient was admitted for intravenous vancomycin therapy, after which her symptoms partially abated.

**Table 1:** Laboratory data at initial presentation.

Variable	Value on Presentation	Reference Range
Hemoglobin (g/dL)	13.3	12.0 - 16.0
White Blood Cell Count (10⁹/L)	10.4	3.5 - 11.0
Platelet Count (10⁹/L)	382	150 - 400
Erythrocyte Sedimentation Rate (mm/h)	6	≤ 12
C-Reactive Protein (mg/L)	4.1	0.0 - 5.0
Sodium (mmol/L)	138	136.0 - 146.0
Potassium (mmol/L)	4	3.5 - 5.1
Bicarbonate (mmol/L)	28.8	22.0 - 32.0
Creatinine (mg/dL)	0.55	0.51 - 0.95
Alanine Transaminase (U/L)	10	≤ 35

**Table 2:** Antibiogram of MRSA cultured from a bronchoalveolar lavage specimen. MRSA: methicillin-resistant Staphylococcus aureus; MIC: minimum inhibitory concentration

Antibiotic	MIC (mg/L)	Interpretation
Clindamycin	≤0.25	Sensitive (S)
Linezolid	1	Sensitive (S)
Oxacillin	>2.0	Resistant (R)
Penicillin	>0.25	Resistant (R)
Vancomycin	1	Sensitive (S)

**Figure 2:**
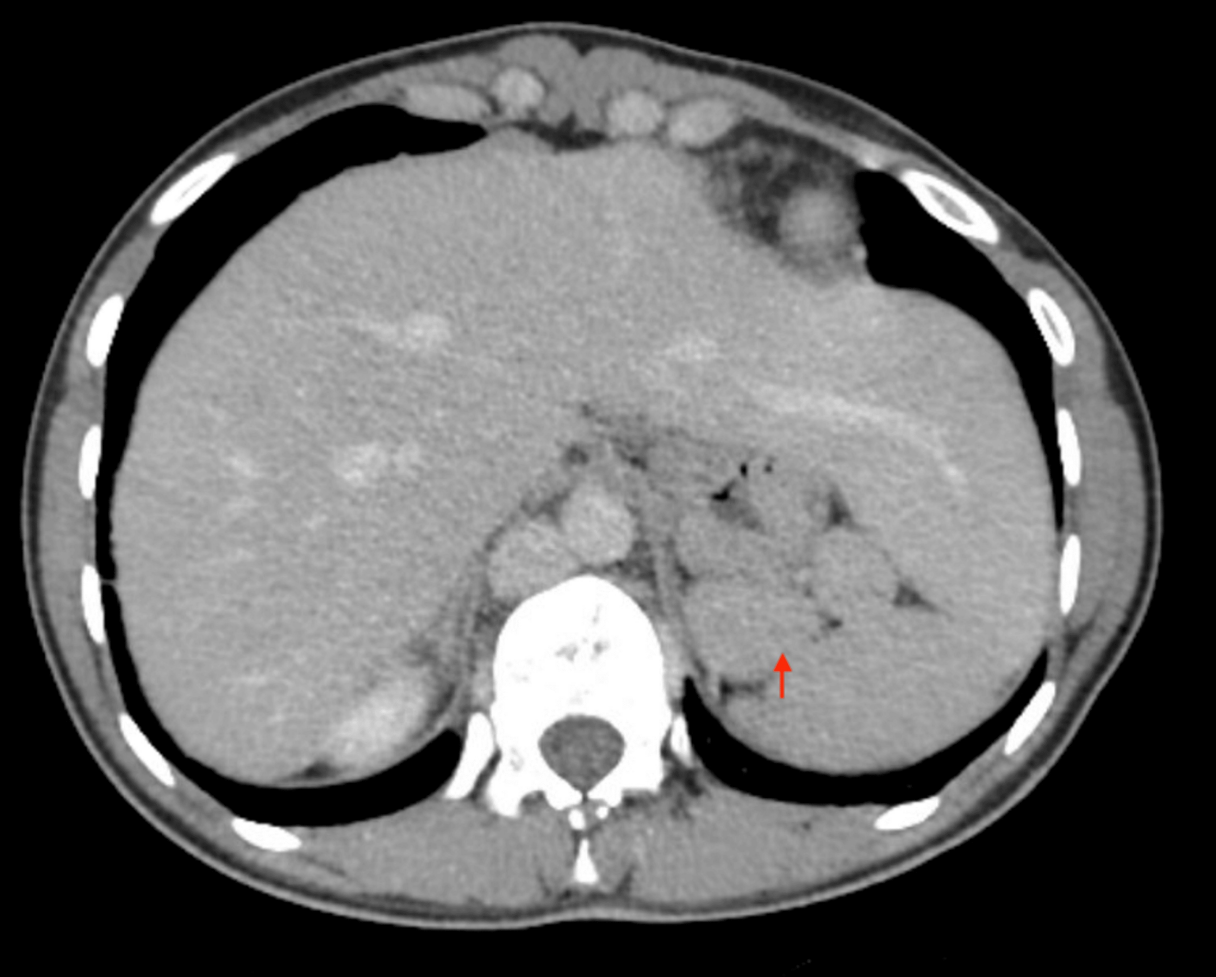
Abdominal CT scan demonstrating the presence of multiple spleens (red arrow) and a liver extending far to the left side.

After completion of antibiotic therapy, a CT scan of the sinuses was performed. It was remarkable for bilateral frontal sinus aplasia, hypoplasia of the maxillary and ethmoidal sinuses with reactive mucosal hypertrophy, absence of the uncinate processes, and signs of middle ear infiltration. A previous abdominal CT scan was reviewed, which showed evidence of an enlarged left liver, a multinodular spleen, distal pancreatic atrophy, an interrupted inferior vena cava, and drainage of the hepatic veins into the right atrium (Figure 2). Electrocardiogram and transthoracic echocardiography findings were unremarkable.

A nasal biopsy was performed and revealed an absence of ciliary motility. Transmission electron microscopy showed inner dynein arm defects, with a significant portion having central apparatus defects, without outer dynein arm defects. Genetic testing by exome sequencing based on a gene panel of 148 genes known to be associated with heterotaxy syndrome and PCD was conducted and detected a novel homozygous c.2347_2351del (p.Phe783ThrfsTer3) PVS1 null variant in exon 17 of the *CCDC39* gene. A diagnosis of autosomal recessive primary ciliary dyskinesia-14 with associated heterotaxy syndrome was made. The patient and her partner were referred for genetic counseling, and it was decided, given their plans for children, that genetic testing would be beneficial to determine if her partner carried any relevant genetic variations.

## Discussion

One of the most prominent features of heterotaxy syndrome in patients presenting without cardiac abnormalities is an aberrancy of the spleen or other unusual arrangements of the intra-abdominal organs. Heterotaxy is defined as an abnormality in which the internal thoracoabdominal organs demonstrate an abnormal arrangement across the left-right axis of the body in different presentations. Although humans show symmetry in their external features, the usual physiological arrangement of a human's visceral organs shows a natural form of asymmetry, whereas, in patients with heterotaxy, there is a remarkable form of unexpected symmetry in some organs to variable degrees. The two extreme forms of presentation can be situs solitus and situs inversus, which are mirror images of each other. The main driver of this asymmetry is a process of lateralization during the embryonic stages of development, where the midline cells play a crucial role in directing the structures to their appropriate positioning in the body. The intricacies of this process are not completely understood, but an important role is fulfilled by the dynein arms of the cilia on notochordal cells [1]. 

Mutations in genes such as *CCDC39*, which encode essential components of the ciliary dynein arms, can disrupt this lateralization process, leading to heterotaxy and associated phenotypes. These mutations impair the structural integrity and function of motile cilia, highlighting their critical role in generating nodal flow during embryogenesis. The role of cilia in the development of left-right asymmetry during embryogenesis is a fascinating and complex process. Research in murine models with ciliary abnormalities has significantly advanced our understanding, demonstrating that motile cilia are indeed present on the embryonic node, an organizer structure crucial for establishing asymmetry. These cilia, driven by dynein-mediated movement, produce a clockwise motion that generates a robust leftward flow of extraembryonic fluid, known as nodal flow. This leftward nodal flow initiates a cascade of asymmetrical signals that orchestrate critical processes such as the D-looping of the primitive heart tube and the asymmetrical morphogenesis of cardiac structures [2]. 

In the presented case, a novel mutation in CCDC39 was identified, linking the patient’s heterotaxy syndrome with PCD. The patient exhibited chronic cough, dyspnea, and recurrent sinopulmonary infections. These clinical manifestations reflect impaired ciliary motility, confirming the functional consequences of the genetic mutation. Furthermore, incidental imaging findings revealed multiple spleens, consistent with polysplenia, a hallmark of left isomerism. 

Mutations affecting ciliary function, particularly in the axonemal dynein complex, can disrupt this process, leading to heterotaxy or randomization of left-right organ positioning. Interestingly, the overlap between PCD and heterotaxy is further supported by findings in other murine models, such as those with mutations in *DNAH5*, a dynein heavy chain gene. These mutations result in features consistent with PCD, including chronic respiratory infections and situs randomization, with some *DNAH5*-mutant mice also displaying heterotaxy. These observations underscore the intricate link between the functional integrity of motile cilia, the establishment of left-right asymmetry, and the clinical manifestations of both heterotaxy syndrome and PCD [3]. 

In a complex process, such as lateralization, problems can occur at any stage to any degree. As said, there are two extremes of this spectrum, but there is a wide range of variable presentations of situs ambiguus. The most debilitating consequence is usually an abnormal arrangement of the heart, particularly the atrial appendages. A commonly used substratification is left versus right isomerism. Isomerism is defined as a condition in which morphologically right or left structures are found on the same side of the body [4]. 

Right isomerism is usually more severe and has higher mortality and morbidity rates, with a high percentage of affected patients dying at birth or requiring neonatal surgery. This is mostly due to more pronounced cardiac abnormalities. There usually is a degree of abnormal cardiac positioning, associated with anomalous pulmonary venous circulation, atrioventricular septal defects, double outlet right ventricle, pulmonary atresia or stenosis, an absent coronary sinus, or a predilection to tachyarrhythmias due to the presence of bilateral sinoatrial or atrioventricular nodes. The lungs may display a bilateral trilobar organization [5]. 

On the other hand, patients who predominately have left isomerism usually have milder cardiac manifestations that can go undetected at birth or in their early life. Although these conditions are not identical, left isomerism is commonly referred to as polysplenia. They can also present with abnormal cardiac positioning, anomalous pulmonary venous circulation, and atrioventricular septal defects. A more common conduction aberrancy is the morphological or functional absence of the sinus node, resulting in slow atrial or junctional rhythms, or complete heart block [6]. The lungs can be bilaterally bilobar. Clinically relevant, however, is that in almost all cases of heterotaxy syndrome, there is an aberrancy of the spleen, and such a finding should raise awareness to investigate for other associated anomalies. Recognition of these anomalies provides important data on the possible associated comorbidities. Knowledge of anatomical variations in the venous circulation and abdominal organs will prove useful in case of future need for interventional procedures to mitigate periprocedural complications. As previously stated, the course of patients with left isomerism is usually milder. An increasing number of patients are being diagnosed in adulthood by the incidental discovery of polysplenia on cross-sectional or ultrasound imaging. The second most common abnormality is an interruption of the inferior vena cava with azygous continuation. Involvement of the pancreas is usually due to dorsal pancreatic agenesis or, to a lesser extent, there might be an annular pancreas. Patients affected with this may be more prone to pancreatitis or diabetes mellitus [7]. 

The nature of cardiac disease is the most important prognostic factor for survival; however, a good understanding of other affected systems will lead to earlier detection and management of possible complications. A wide range of extracardiac manifestations has been reported. The most easily recognized are usually gastrointestinal in nature. Intestinal malrotation with pronounced hepatomegaly is easily noted in the imaging studies that detected polysplenia in the first place. In such cases, meticulous care should be given to detect abnormalities of the pancreas and venous circulation of the liver. An interrupted inferior vena cava is the second most common extracardiac abnormality, but a wide range of anatomical variations in the portal vein have been reported as well. The genitourinary system is affected in approximately a quarter of patients, with abnormalities ranging from bilateral cryptorchidism to structural kidney and ureteral derangements [8]. 

In this patient, polysplenia was identified alongside an interrupted inferior vena cava with azygous continuation. These findings aligned with typical left isomerism, further substantiating the heterotaxy diagnosis. While no cardiac anomalies were identified, the patient’s recurrent respiratory symptoms necessitated a broader investigation, ultimately leading to the discovery of PCD. The dual involvement of structural and functional abnormalities highlights the need for comprehensive diagnostic evaluations in heterotaxy patients. 

It can be concluded that structural abnormalities, on the one hand, are rather large and obvious, and on the other hand, some small and intricate, are the most common features in patients with heterotaxy syndrome. The aim of this case report, however, is to draw attention to the functional abnormalities associated with the condition. 

Morphological splenic aberrations, be it the more obvious asplenia or the hypofunctional spleens in patients with polysplenia, render them more vulnerable to infections, especially with encapsulated organisms. As there is still intact splenic tissue in patients with polysplenia compared to patients with complete absence, functional capacities are usually reduced. Rapid recognition of infections and adequate prevention with vaccination are warranted. As mentioned earlier, their structural abnormalities make them more prone to infection at specific sites such as urinary tract infections [9]. 

How the respiratory tract is affected remains an under-recognized fact. However, as briefly alluded to before, when the cause of abnormal lateralization of the internal organs is considered to be an abnormality in the cilia that drives this process, it might not be surprising that there exists a lot of overlap between heterotaxy syndrome and primary ciliary dyskinesia or Kartagener syndrome. Common features include recurrent sinopulmonary infections, bronchiectasis, and atelectasis due to poor secretion clearance because of ciliary dysfunction. Better awareness of these features should result from an increased understanding of the genetics and etiological drivers of heterotaxy syndrome. This should, in turn, lead to better detection of the possibly associated structural abnormalities in patients presenting with features of ciliary dysfunction where there is no prior knowledge of their internal anatomy [5]. 

This patient’s case underscores the necessity of investigating ciliary dysfunction in heterotaxy syndrome, even in the absence of overt cardiac defects. The novel *CCDC39 *variant emphasizes the genetic contribution at play in these overlapping syndromes and demonstrates the need for targeted diagnostic strategies. Improved recognition of PCD in the context of heterotaxy can prevent long-term complications through earlier intervention and comprehensive care. 

As stated before, there is an intricate mechanism in the early embryo that establishes the physiological asymmetry of the internal organs. Different pathways are involved in this process, including intracellular signaling mechanisms, the formation of motile and sensory cilia, intraflagellar transport, and gene expression. Several genetic defects have been described involving one or more of these steps, with the result being a form of left-right asymmetry [10]. These can be monogenic mutations, chromosomal derangements, or as part of a genetic syndrome. Syndromes involving dysfunction of cilia are the best understood of these syndromes. The novel mutation discovered in this patient was involved in the *CCDC39 *gene responsible for the production of a protein necessary for inner dynein arm attachment of cilia [11].

## Conclusions

The case presented here portrays an example of a patient in whom several signs during childhood and adolescence, such as persistent respiratory infections, chronic cough, polysplenia, and hepatomegaly, may have hinted at the presence of an underlying disorder. Better awareness and understanding of the conditions associated with either heterotaxy syndrome or primary ciliary dyskinesia should lead to earlier recognition of these patients in the future, which in turn will lead to a more timely diagnosis, appropriate interventions, and improved patient outcomes. Although the wide heterogeneity of these conditions makes it difficult to offer a standardized approach, once suspected, an appropriate multidisciplinary approach should be adopted. The team should include pulmonologists and otolaryngologists to address upper and lower respiratory complications, cardiologists to assess for any subtle or latent cardiac abnormalities, gastroenterologists to evaluate gastrointestinal and hepatic anomalies, and immunologists to address potential splenic dysfunction and heightened infection risk. Geneticists and genetic counselors play a pivotal role in confirming the diagnosis, identifying familial patterns, and offering guidance on potential future implications. By adopting this coordinated and comprehensive approach, clinicians can ensure appropriate follow-up, management of comorbidities, and prevention of further complications, ultimately improving the quality of life for affected patients.
